# Transcriptional co-regulator OCA-B/Pou2af1 restricts Th2 differentiation

**DOI:** 10.3389/fimmu.2025.1548636

**Published:** 2025-04-29

**Authors:** Erik P. Hughes, Asit K. Manna, Wenxiang Sun, Sandra M. Osburn-Staker, Samuel Aamodt, Kristi J. Warren, James E. Cox, Dean Tantin

**Affiliations:** ^1^ Department of Pathology, University of Utah School of Medicine, Salt Lake City, UT, United States; ^2^ Huntsman Cancer Institute, University of Utah School of Medicine, Salt Lake City, UT, United States; ^3^ Department of Biochemistry, University of Utah School of Medicine, Salt Lake City, UT, United States; ^4^ Department of Internal Medicine, University of Utah School of Medicine, Salt Lake City, UT, United States

**Keywords:** CD4+ T cells, Th2, OCA-B, Bob1, *Pou2af1*, *Gata3*

## Abstract

**Background:**

Type 2 immunity is initiated through a synergistic response between innate and adaptive immune cells to facilitate host-pathogen defense and wound repair, yet aberrant responses can contribute to chronic inflammation and allergic disease. CD4^+^ type 2 helper T (Th2) cells facilitate the adaptive immune response through the secretion of cytokines such as IL-4, IL-5, and IL-13. While the Th2 program is governed by the transcription factor GATA3, less is known about regulators that fine-tune the Th2 cytokine response.

**Method:**

We used a proximity labeling system to map proteins associated with the transcriptional co-regulator OCA-B, encoded by *Pou2af1*, in T cells. We used a series of genomic, biochemical and immunological assays to probe the interaction with one particular hit from the screen.

**Results:**

We find that OCA-B indirectly associates with GATA3. ChIP-seq analysis reveals coenrichment of Gata3 and the transcription factor Oct1, a partner protein of OCA-B, at genomic locations responsible for the Th2 program including *Il4, Il13, Il5, Gata3*, and *Irf4*. DNA binding data using recombinant proteins and reporter data using T cell lines are consistent with a model in which OCA-B restricts transcription at the Th2 locus control region and subsequent IL-4 and IL-13 secretion. Finally, in an *in vivo* papain allergy model we show OCA-B expression in T cells limits the frequency of T cells within the lung.

**Conclusion:**

These findings shown that OCA-B helps restrict Th2 function at least in part through communication with GATA3.

## 1 Introduction

Upon stimulation, naïve CD4^+^ T cells can differentiate into various T helper (Th) cell subsets tailored to combat specific pathogens. These subsets include the Th1, Th2, Th9, Th17, Tfh, and Tregs (1). Each subset is defined by the effector cytokines they produce and the transcriptional machinery that enforce the Th subset identity (2–4). For example, Th1 cells are characterized by the expression of the transcription factor T-bet and the secretion of IFNγ, while Th2 cells express the transcription factor Gata3 and secrete IL-4, IL-5, and IL-13 (2, 3). As a given Th lineage is established, lineage-inappropriate genes are silenced such that other Th lineages are suppressed (5–8). Following initial observations that chromatin structure changes occur near *Ifng*, *Il4*, and *Il13* as naïve CD4^+^ T cells differentiate into Th1 or Th2 cells (9), numerous reports have highlighted DNA regulatory elements, long-range DNA interactions, transcription factors, and co-regulatory factors that influence T cell fate decisions (6, 10, 11). An example is the transcription factor Oct1 (encoded by *Pou2f1*), which together with CTCF mediates interchromosomal associations between the Th2 locus control region or LCR and the *Il17a/f* locus (12). Disruption of this association leads to Th17 biased cell fate, suggesting interchromosomal associations organized by Oct1/CTCF restrict Th17 differentiation (12). In naïve T cells, Oct1 associates with the repressive chromatin remodeling complex NuRD, to retain target gene methylation and limiting transcription (13). Following T cell stimulation, Oct1 dissociates from the NuRD complex and instead associates with the transcriptional coregulator OCA-B (*Pou2af1*) and the histone demethylase Jmjd1a to remove inhibitory histone methylation marks at target genes, thereby allowing for robust expression upon restimulation (13, 14). These data suggest that Oct1 assists in CD4^+^ T cell fate by controlling lineage-specific gene expression. OCA-B regulates Th17 polarization (15), however the role of T cell OCA-B in the regulation of other lineages is unknown.

Here we investigate the role of OCA-B in Th2 lineage fate decisions. Through a biotin ligase proximity labeling system in human SupT1 T cells, we identify prospective associations between OCA-B and proteins such as GATA3, JMJD1C, MCRIP1, and multiple components of the SWI/SNF complex. Investigating the observed proximity interaction between OCA-B and Gata3, we re-analyzed published ChIP-seq data for both proteins, observing that they are coenriched more than what would be expected by chance. Example include prototypic Th2 genes such as *Rad50, Gata3*, and *Irf4*. OCA-B destabilizes GATA3 DNA binding *in vitro* and suppresses gene expression from an *Il4* regulatory element in reporter assays. Consistent with these findings, Th2 differentiation of OCA-B deficient naïve CD4^+^ T cells indicates that OCA-B restricts IL-4 and IL-13 cytokine production. Finally, we find that *in vivo* allergen challenge of mice lacking T cell OCA-B increases frequencies of T cell infiltration in the lung.

## 2 Methods

### 2.1 Cell lines and animals

SupT1 T cells (ATCC, CRL-1942) and derived clones were grown in complete media (RPMI 1640, 10% fetal bovine serum, 1% penicillin/streptomycin, 2 mM GlutaMAX). OCA-B T cell conditional knockout mice (*Pou2af1^fl/fl^*;CD4-Cre) and littermate flox-only controls have been reported previously (16) and were pure C57BL/6 background. All animal experiments conducted in this study were carried out in accordance with protocols by the University of Utah Institutional Animal Care and Use Committee.

### 2.2 OCA-B isoform construct generation

Wild-type *Ocab*, *Ocab*-p34-G/A, and *Ocab*-p35-M/S cDNA were amplified from parent pcDNA3.1 plasmids (17)(a gift from Robert Roeder, Rockefeller University, New York, NY) and cloned into the pMIGR1 retroviral vector as previously described (18). In-frame *Ocab*-BirA fusion proteins were generated by subcloning wild-type, p34, and p35 *Ocab* isoforms from pMIGR1 vectors into the MCS-BioID2-HA vector (19). Site directed PCR mutagenesis was used to introduce a p40 (CTG to ATG) mutation and the generation of *Ocab-*p35-ATG-M/S and *Ocab*-p40-ATG-G/A-M/S constructs. cDNA was amplified from wild-type and isoform enriched *Ocab* constructs and subcloned into the pLVX-Tight-Puro vector (Clontech Laboratories). Primers for amplification were as follows: wild-type *Ocab-*BirA-Fwd(5’- AAATATGCGGCCGCCTGTCTGCTTCAAAGAGAAAAG-3’), p40-ATG-*Ocab*-BirA-Fwd(5’- AAATATGCGGCCGCATGTCTGCTTCAAAGAGAAAAG-3’), and BirA-Rvs (5’- CCGGAATTCCTATGCGTAATCCGGTACA-3’).

### 2.3 Biotin IDentification proximity labeling

Tet-On SupT1 cells were generated by transduction with a Tet-On lentiviral vector (Lenti-XTM Tet-On Advanced, Clontech Laboratories, Inc) and selection with 500 μg/mL G418. Tet-On SupT1 cells were subsequently transduced with either empty vector (EV), BirA, or wild-type, p34, p35, or p40 isoform-enriched Ocab-BirA virus (pLVX-Tight-Puro, pLVX-Tight-Puro-BirA-HA, or pLVX-Tight-Puro-Ocab-wt-HA, pLVX-Tight-Puro-Ocab-G/A-HA, pLVX-Tight-Puro-Ocab-ATG-M/S-HA, or pLVX-Tight-Puro-Ocab-ATG-G/A-M/S-HA respectively) and selected with 0.5 μg/mL puromycin to generate control (EV and BirA only) and isoform Ocab-BirA fusion cell lines. Triplicate control and Ocab isoform cell lines were cultured, lysed, and processed as previously described (19) before being subjected to mass spectrometry.

### 2.4 Mass spectrometry

Triplicate control and Ocab isoform protein samples were processed for mass spectrometry as previously described (20). Briefly, proteins were reduced and digested with trypsin. Peptides were extracted and subjected to reverse-phase nano-LC/MS/MS. MS/MS data were compared to the UniProt *Homo sapiens* taxonomy database. Protein abundance and replicate variance for each sample was evaluated using Proteome Discoverer (ThermoFisher).

### 2.5 SupT1 CRISPR/Cas9 OCA-B mutation

Guide RNAs (gRNAs) targeting human *POU2AF1* exons 2, 3, and 4 were as follows: hOCAB gRNA1: TTCACACGGACGCCCTGGTATGG, hOCAB gRNA2: TACTCGGTGTAAGGTGTCCATGG, hOCAB gRNA3: CCTGGCGACCTACACCACAGTGG. gRNAs were combined 1:1:1 (67μM each) and added to an equal volume of ATTO-550 TracrRNA (100μM, Integrated DNA Technologies). The mixture was incubated at 95°C for 5 min then cooled to RT for 20 min. An equal volume of Cas9 (Macro Labs, Berkeley) was added to the annealed RNA mixture and incubated at RT for 20 min. 1 μL RNA/Cas9 mixture was added to 2.0×10^5^ SupT1 cells in 9 μL buffer T (ThermoFisher), and the suspension electroporated using a Neon device (ThermoFisher, 1700V/10MS/3pulse). Following electroporation, ATTO-550^+^ cells were isolated using a FACSAria (BD Biosciences) and cultured in complete media for 48 hrs. Following electroporation recovery, cells were single cell-cloned by limiting dilution and assessed for OCA-B expression by immunoblot.

### 2.6 Co-immunoprecipitation

1.0×10^7^ Parental SupT1 and derived hOCA* cells lacking a large portion of OCA-B were lysed in lysis buffer (50 mM Tris pH 7.5, 500 mM NaCl, 0.5 mM EDTA, 1% Triton X-100) containing the same protease inhibitors as above, and rotated at 4°C for 20 min. Lysates were clarified by centrifugation (13,000×*g*) for 10 min at 4°C. 2 µg of primary antibodies were added to the lysates, which were rotated at 4°C overnight. Antibodies used were as follows: anti-Oct1 (12F11, Santa Cruz sc-8024), anti-GATA3 (D13C9, Cell Signaling 13411), anti-OCA-B (E5K1D, Cell Signaling 43079). Isotype control IgG was purchased from Jackson ImmunoResearch. Protein G Dynabeads (ThermoFisher) were washed with lysis buffer and added to the cell lysis-antibody mix and subsequently samples were rotated at 4°C for 5 hr. Protein-bound Dynabeads were then washed with 500 µl lysis buffer for 5 times, and proteins were eluted with 40 µL 2x SDS loading buffer by heating at 98°C for 8 min.

### 2.7 ChIP-seq

Previously published Oct1 (35 million, GSM1611108) and Gata3 (8 million, GSM742023) single-end reads were realigned to the *mm39* genome using Novaalign V4.03.01 (Novocraft). Aligned Oct1 and Gata3 data was analyzed using Macs2 (21), with *q* < 0.01, minimum peak size 250 bp and max-gap 100 bp. Overlapping peaks were defined as <100 bp between peak summits. Relative distance comparison analysis was conducted using *reldist* (BEDTools, v2.31.0) (22).

### 2.8 Oct1, OCA-B and GATA3 purification

The Oct1 DNA binding domain was amplified from pBabe-hOct1 (23) by PCR using the primers 5**’**- GGCTCGAGGAGAGGAGCCCAGTGACCTTG-3’, 5**’**-GGATCCTTTTTCTTTCTGGCGGCG-3’ and cloned into the pET28a vector using *Xho*I and *Bam*HI sites. OCA-B with C-terminal twinstrep and FLAG tags was cloned into the pACE-MAM2 vector (Geneva Biotech) using the primers 5**’**- GGATCTCGAGCCATGCTCTGGCAAAAACCC-3’, 5**’**-CCACGCAGAATGCATAAAGCCTTCCACAGAGAGAG-3’. The GATA3 expression plasmid was purchased from AddGene (Cat #1419). The Oct1 DNA binding domain was expressed and purified from Rosetta 2(DE3) pLysS cells (Sigma) using Histrap columns (Sigma). OCA-B and GATA3 expression plasmids were transfected into Expi293F cells (ThermoFisher) using the ExpiFectamine 293 Transfection Kit (ThermoFisher) following the manufacturer instructions. 48 hr post-transfection, cells were harvested and washed with ice-cold PBS. Overexpressed proteins were purified using anti-FLAG (M2)-agarose beads (Sigma) following the protein purification protocol as described in Shen et al. (24).

### 2.9 Electrophoretic mobility shift assays

EMSA was performed using Cy-5-labeled dsDNA probes following published protocols (25). Probes were Octamer-Cy5 (5’-GAGTCCTGGCGGATGCAAATGGTGCTGCTTCG-3’, 5’-CGAAGCAGCACCATTTGCATCCGCCAGGACTC-3’), RHS5_12-Cy5 (5’-TCGAGAAGCGCTGATTAGCATCTGTCATTA-3’, 5’-TAATGACAGATGCTAATCAGCGCTTCTCGA-3’) and RHS6-Cy5 (5’-CACCATGCAAAGGATGTGCGCGGACTCCCTTCCATTTGCTGGCCTCTTATCTGATA-3’, 5’-TATCAGATAAGAGGCCAGCAAATGGAAGGGAGTCCGCGCACATCCTTTGCATGGTG-3’). Briefly, reactions were assembled on ice in 0.6X buffer D (1X buffer D: 20 mM HEPES, 100 mM KCl, 1 mM DTT), containing 50 ng/µl poly-dI/dC, and 1 µg/µl BSA, kept on ice for 15 min, and resolved using 6% native PAGE in a 0.5X TBE buffer. Probes were used at 50 nM and 1 nM for competition experiments. Images were taken using a Molecular Dynamics Typhoon system.

### 2.10 NanoLuciferase

Gene blocks (Integrated DNA Technologies) were synthesized harboring the previously described RHS5_12 (26) (5’-TCGAGAAGCGCTGATTAGCATCTGTCATTA-3’) containing both Oct1 and GATA3 DNA binding motifs (underlined) or mutated motifs (GTAGTTTG or ACTGCA) upstream of a minimal CMV promoter. A CMV-only construct was also synthesized as a control for basal nano-luciferase expression. Gene blocks were subcloned into the multiple cloning site of the pNL2.3 (Promega, Cat# N108A). Parental pNL2.3 was used as an control. Parental SupT1 and hOCA* cells were electroporated using Neon (ThermoFisher, 1700V/10MS/3pulse) with either EV, CMV-only, Oct1^wt^-GATA^wt^, Oct1^wt^-GATA^mut^, Oct1^mut^-GATA^wt^, or Oct1^mut^-GATA^mut^ construct. nLuc expression was evaluated 24 hrs after electroporation using the Nano-Glo system (Promega) on a Modulus microplate reader (Turner Biosystems).

### 2.11 Naïve CD4^+^ T cell isolation and Th2 differentiation

Spleens were isolated from 8 week-old male/female *Pou2af1^fl/fl^* and *Pou2af1^fl/fl^*;CD4-Cre mice by mechanical dissociation and passage through a 70 μm mesh strainer. Erythrocytes were lysed by ammonium-chloride-potassium (ACK) buffer 150 mM NH_4_Cl, 10mM KHCO_3_, 0.1 mM Na_2_EDTA). Naïve CD4^+^ T cells were purified from splenocytes using negative magnetic selection per manufacturer’s protocol (Miltenyi Biotec). For Th2 differentiation, 1.0x10^5^ naïve CD4^+^ T cells/well were seeded in 96-well plates with T-activator CD3/CD28 beads (ThermoFisher) in complete media supplemented with 10 μg/mL anti-IFN-γ antibodies, 10 ng/mL recombinant IL-2 (20U/mL Roche hIL-2), and 10 ng/mL recombinant mIL-4 (Peprotech) for 5 days. Differentiated Th2 cells were analyzed by flow cytometry and qPCR.

### 2.12 Flow cytometry

Cells were cultured in complete media supplemented with 1 μL/mL brefeldin A (Golgiplug, BD), 50 ng/mL phorbol myristate acetate (Sigma-Aldrich) and 1 μg/mL ionomycin (Sigma-Aldrich) for 4 hr. Cells were stained for viability with either Zombie Violet (Biolegend) or Live/Dead Blue (ThermoFisher) and stained for surface markers including CD45-Percp (30-F11, Biolegend), Ly6G-PB (1A8, Biolegend), F4/80-APC (QA17A29, Biolegend), CD64-BV711 (X54-5/7.1, Biolegend), CD11c-PercpCy5.5 (N418, Biolegend), CD11b-APC/Cy7 (M1/70, Biolegend), IA/IE-BUV805 (M5/114.15.1, BD), SiglecF-FITC (S17007L, Biolegend), NK1.1-PECy5 (PK136, Biolegend), CD19-BUV661 (1D3, BD), CD3e-BV605 (145-2C11, Biolegend), CD4-BUV395 (GK1.5, BD), and CD8a-BUV737 (53-6.7, BD). Following surface staining, cells were fixed using cytofix/cytoperm (BD) according to manufacturer’s protocol and stained intracellular for cytokines in BD perm/wash buffer using antibodies against IFNγ-PECy7 (XMG1.2, ThermoFisher), IL-4-PE (11B11, BD), and IL-13-APCeFluor780 (eBio13A, ThermoFisher). Samples were profiled using Cytek Aurora and analyzed using FlowJo software (BD).

### 2.13 qPCR

Total RNA was isolated from differentiated Th2 cells (*Pou2af1^fl/fl^* n=4, *Pou2af1^fl/fl^*;CD4-Cre n=4) using a Quick-RNA MicroPrep Kit (Zymo) per manufactures instructions. cDNA reverse transcription was performed using the SuperScript III First-Strand synthesis kit (ThermoFisher) per manufactures instructions. TaqMan qPCR (Cat#4444556, ThermoFisher) was performed per manufactures directions in triplicate assessing expression of *Il4* (Mm00445259_m1, ThermoFisher), *Il5* (Mm00439646_m1, ThermoFisher), *Il13* (Mm00434204_m1, ThermoFisher), and *Actb* (Mm00607939_s1, ThermoFisher) on a CFX96 Real-Time system (BioRad). Average triplicate Cq values for *Il4*, *Il5*, and *Il13* were normalized to *Actb* and compared between groups.

### 2.14 Papain challenge

10 week-old *Ocab^fl/fl^* and *Ocab^fl/fl^*;CD4-Cre littermate mice were anesthetized by isoflurane inhalation and intranasally challenged with 25 μg papain (ThermoFisher) in 10μL of PBS or saline (Ocab^fl/fl^ papain n=5, Ocab^fl/fl^;CD4-Cre papain n=7, Ocab^fl/fl^ saline n=4, Ocab^fl/fl^;CD4-Cre saline n=4). Mice were treated with either papain or saline on days 0, 1, 2, 7, 8, 9, and 10 then sacrificed on day 11.

### 2.15 Lung isolation

Lungs were perfused with ice cold PBS until white, then removed from the thoracic cavity as described (27). The left bronchus was clamped, and left lung removed and stored on ice in complete media until processing for flow cytometry. The right lung lobes were then inflated with 10% neutral buffered formalin by tracheal instillation, then transferred to an embedding cassette and stored in 10% neutral buffered formalin for 48 hr until histological processing. Right lung lobes were paraffin embedded, and sections cut and stained with hematoxylin and eosin (H&E) by the Associated Regional and University Pathologists (ARUP) at the University of Utah. The left lung was transferred to RPMI 1640 with 2 mg/mL Dispase II (Sigma-Aldrich) and finely minced with a sterile surgical scalpel. Tissue was digested at 37°C for 1 hr, passed through a 70 μm mesh strainer, and processed for flow cytometry.

### 2.16 Histopathology

Paraffin-embedded right lung lobe sections were cut and stained with hematoxylin and eosin (H&E) by the Associated Regional and University Pathologists (ARUP) at the University of Utah. Blinded histological scoring was performed to determine % of pulmonary veins with infiltrate × measure number of cellular depth of perivenous infiltrate. Images were taken at 20x on a Lecia DM2500 microscope with an IMI Tech IMC 4050FT camera.

## 3 Results

### 3.1 OCA-B isoform proximity labeling identifies GATA3 as a proximal protein

**Figure 1:**
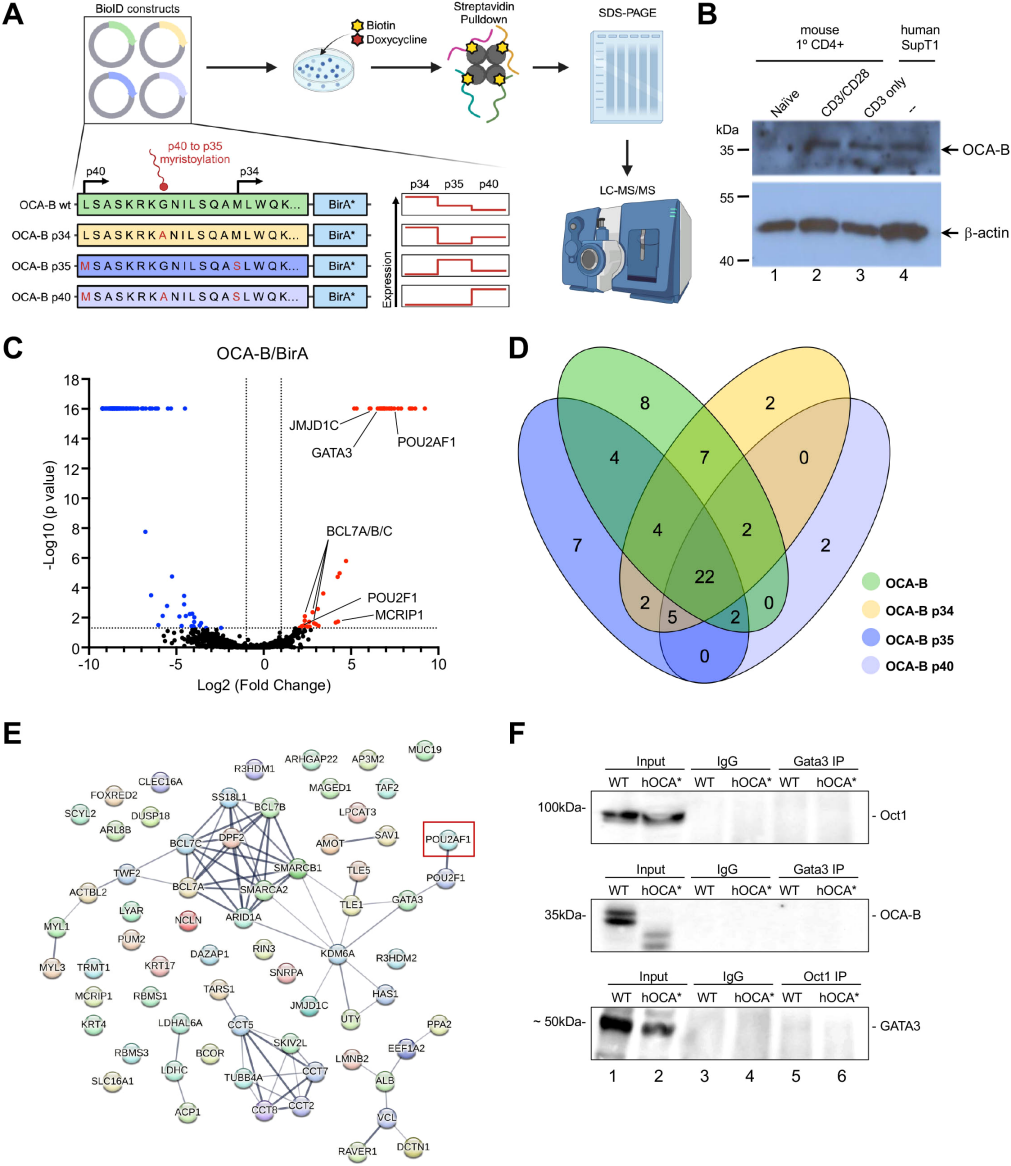
OCA-B isoform proximity labeling identifies GATA3 as a consistently proximal protein. **(A)** Schematic showing the OCA-B isoform constructs and their mutations used to generate OCA-B-BirA fusion proteins and which OCA-B isoforms are expressed. OCA-B WT is capable of expressing OCA-B from both the canonical p34 start codon and the non-canonical p40 codon which gets myristoylated generating the p35 isoform. OCA-B p34 construct includes a p35 G to A mutation which prevents myristolation, resulting in only p34 and p40 isoform expression. OCA-B p35 includes two mutations, a p40 L to M mutation and a p34 M to S mutation, stopping p34 isoform expression and increasing p40 expression which gets myristoylated into the p35 isoform. Finally, OCA-B p40 contains three mutations, a p40 M to S, a p35 G to A, and a p34 M to S, which drives expression of the p40 isoform which is unable to be myristoylated. The expression chart shows isoform expression level (red line) for each construct. OCA-B-BirA isoform and BirA-only constructs were integrated into the SupT1 T cell line and then cells were supplemented with doxycycline and biotin to promote fusion protein expression and proximity biotinylation. Isolated protein was subjected to streptavidin pull-down to enrich biotinylated proteins and enriched proteins were analyzed by LC-MS/MS. **(B)** Immunoblot comparing OCA-B protein expression in primary mouse naïve, CD3 stimulated, and CD3/CD28 stimulated CD4^+^ T cells to the SupT1 T cell line. **(C)** Volcano plot depicting proteins identified by mass spectrometry positively and negatively enriched from wild-type OCA-B-BirA (n=3) transduced relative to BirA-only (n=3) transduced control SupT1 cells. Proteins significantly elevated with the wild-type OCA-B replicates are shown in red, and proteins significantly elevated in BirA-only replicates are shown in blue. **(D)** Venn diagram shown the number of proteins significantly elevated over the BirA-only control within each OCA-B isoform and any protein overlap between isoforms. **(E)** String map showing significantly enriched proteins compared to the BirA-only control from all OCA-B isoforms. Connecting line thickness represents the strength of the protein association. OCA-B (POU2AF1) is marked by a red box. **(F)** Coimmunoprecipitation assays evaluating protein-protein interactions between GATA3, Oct1, and OCA-B using SupT1 cell lysates. “WT” indicates normal parent SupT1 cells. “hOCA*” indicates SupT1 cells in which CRISPR was used to disrupt OCA-B, creating a significant truncation.

OCA-B can be translated at canonical and non-canonical start codons to yield 34 and 40 kDa proteins, the latter of which is cleaved and myristylated to generate a p35 isoform (17). To identify isoform-specific OCA-B interactors, we utilized the Biotin Identification (BioID) proximity labeling biotin ligase (BirA) system coupled with subsequent proteomic analysis using mass spectroscopy. Doxycycline-inducible lentiviral constructs were engineered to express in-frame OCA-B-BirA fusion proteins. A wild-type (WT) cDNA capable of expressing all the isoforms (p34, p35, and p40) was used, as well as specific mutations to enrich for the p34, p35, or p40 isoforms (**Figure 1A**). EV and BirA-only control constructs were used to evaluate baseline biotinylation and non-specific proximity labeling. The human SupT1 T cell line expresses endogenous OCA-B at levels similar to activated primary murine T cells (**Figure 1B**). The constructs were transduced into SupT1 cells, and clones expressing different forms of OCA-B comparably were selected for further study. Doxycycline successfully induced expression of the OCA-B BirA fusions, and biotinylation following streptavidin pulldown (PD) were validated by anti-OCA-B and streptavidin immunoblots (**Supplementary Figures 1A–C**).

Biotinylated proteins from triplicate control and OCA-B isoform cell lines were enriched by streptavidin pulldown and analyzed by ultra-high mass range hybrid quadrupole-orbitrap mass spectrometry (**Supplementary Table 1**). Significant protein enrichment in each isoform over the non-specific BirA control was observed (**Figure 1C**, **Supplementary Figures 1D–F**). Enriched proteins within the OCA-B samples included OCA-B itself, the known interactor Oct1 (POU2F1), and multiple novel potential interactions such as GATA3, JMJD1C, MCRIP1, and multiple components of the SWI/SNF chromatin remodeling complex (BCL7 A/B/C, ARID1A, SMARCA2, SMARCB1). While isoform-specific protein enrichments were observed such as the BCL6 corepressor BCOR for p34, the histone lysine demethylases KDM6A and KDM6C for p35, and the actin-binding protein TWF2 for p40, most significant proteins were coenriched amongst all isoforms (**Figures 1D, E**, **Supplementary Table 1**).

### 3.2 Oct1 and GATA3 are coenriched at Th2 lineage commitment genes

**Figure 2:**
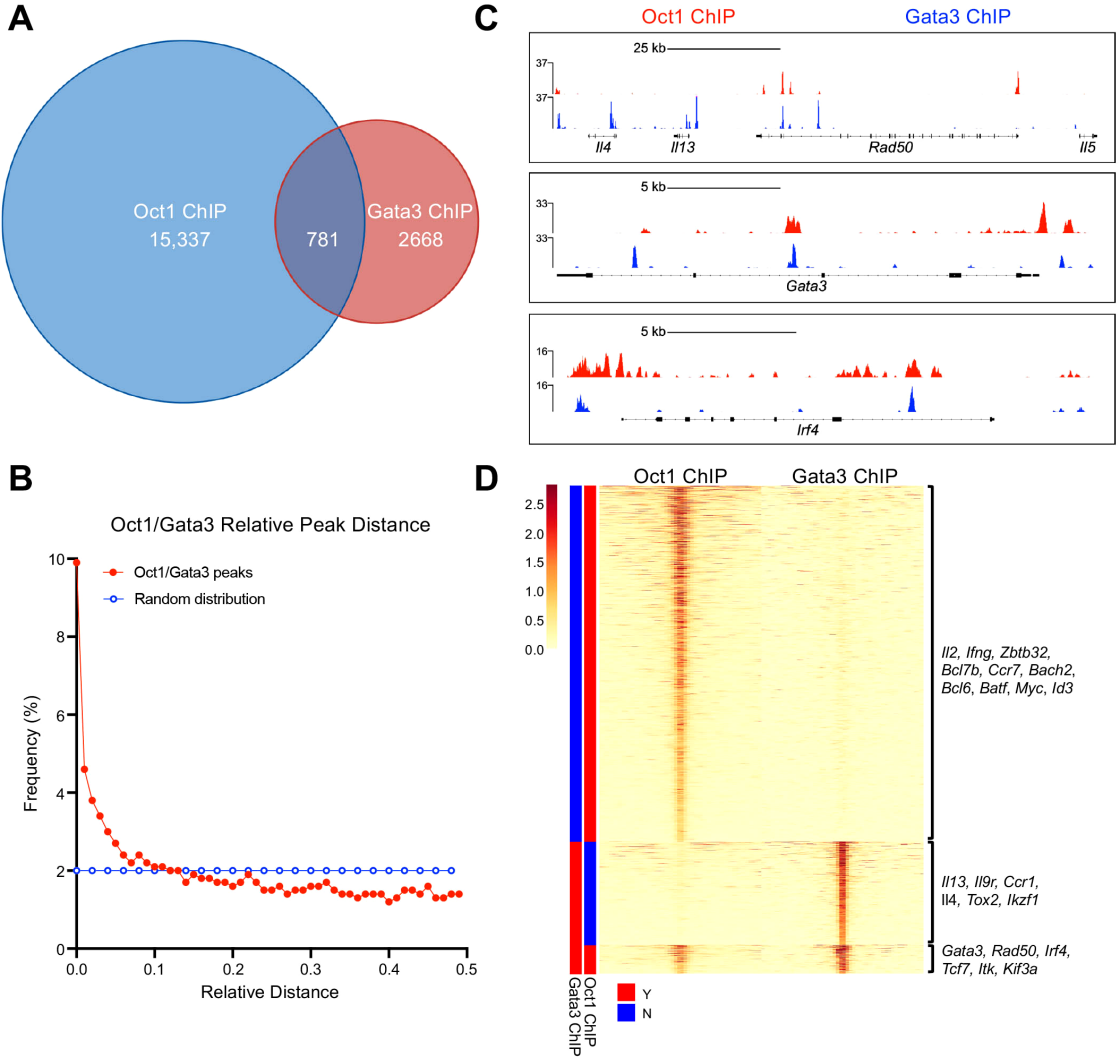
Oct1 and Gata3 are coenriched at Th2 lineage commitment genes. **(A)** Previously published Oct1 and Gata3 ChIPseq datasets were reanalyzed to determine coenrichment. Venn diagram is shown depicting the number of unique Oct1 and Gata3 ChIP peaks and the number of Oct1/Gata3 ChIP peaks that overlapped within 100 bp. **(B)** Spatial correlation comparing the relative abundance of Oct1 and Gata3 peaks based on the distance between them. **(C)** Example genome tracks showing Oct1 and Gata3 ChIP peaks (Th2 locus, *Gata3*, and *Irf4*). **(D)** Heatmap showing hierarchically clustered ChIP peaks aligned by midpoint between Oct1 and Gata3 datasets. Representative gene names are listed for each cluster.

Amongst the coenriched proteins was the Th2 master transcription factor GATA3. Reciprocal Co-immunoprecipitations (CoIP) between Oct1, OCA-B, and GATA3 were performed from cell lysates from SupT1 cells, as well as cells engineered by CRISPR/Cas9 to express a truncated form of OCA-B (hOCA*, **Figure 1F**). No stable Co-IP interaction was observed between either Oct1 and GATA3 or OCA-B and GATA3, suggesting that the BioID enrichment may be due to proximity rather than a physical interaction. To test if Oct1/OCA-B and Gata3 regularly colocalize to similar genomic locations, we compared published activated primary CD4^+^ T cell Oct1 chromatin immunoprecipitation sequencing (ChIPseq (14),) to a Th2 cell Gata3 ChIPseq dataset (28). Following alignment to the murine *GRCm39* genome and quality control, 15,337 Oct1 and 2,668 Gata3 peaks were identified, 781 of which (29% of the Gata3 peaks) overlap (<100bp, **Figure 2A** and **Supplementary Table 2**). Analysis of the relative distance between Oct1 and Gata3 peaks using methodologies described by Favorov et al. (22) identified an increased fraction of Oct1 and Gata3 peaks at shorter distances, indicating a spatial correlation (**Figure 2B**). Investigating individual genome tracts with known Gata3 binding events, we identified Oct1/Gata3 coenrichment at multiple Th2 associated regions including the Th2 LCR at the 3’ end of *Rad50* (RHS5 and RHS6) and 7kb downstream of *Il4*, *Gata3*, and *Irf4* (**Figure 2C**). Hierarchical clustering of the ChIP peak densities indicated that while the majority of Oct1 peaks were unique, a large proportion of Gata3 peaks overlapped with Oct1 peaks (**Figure 2D**). Mapping to the nearest gene showed that unique Oct1 peaks enriched near genes with broad T cell functions such as *Il2*, *Ifng*, *Zbtb32*, *Myc*, and *Bcl6*, while unique Gata3 peaks enrich genes associated with Th2 cell fate and function such as *Il4*, *Il13*, *Il9r*, and *Ccr1* (**Figure 2D**). Interestingly, coenriched peaks enrich near genes with both broad T cell and Th2-specific functions including *Gata3*, the *Rad50* (Th2) LCR, *Irf4*, *Tcf7*, and *Itk*. Together these findings suggest that Oct1 and Gata3 spatially correlate and are coenriched near multiple Th2 regulatory regions, consistent with a model in which they coregulate Th2 cell fate and function.

### 3.3 OCA-B restricts gene expression at the Th2 LCR

**Figure 3:**
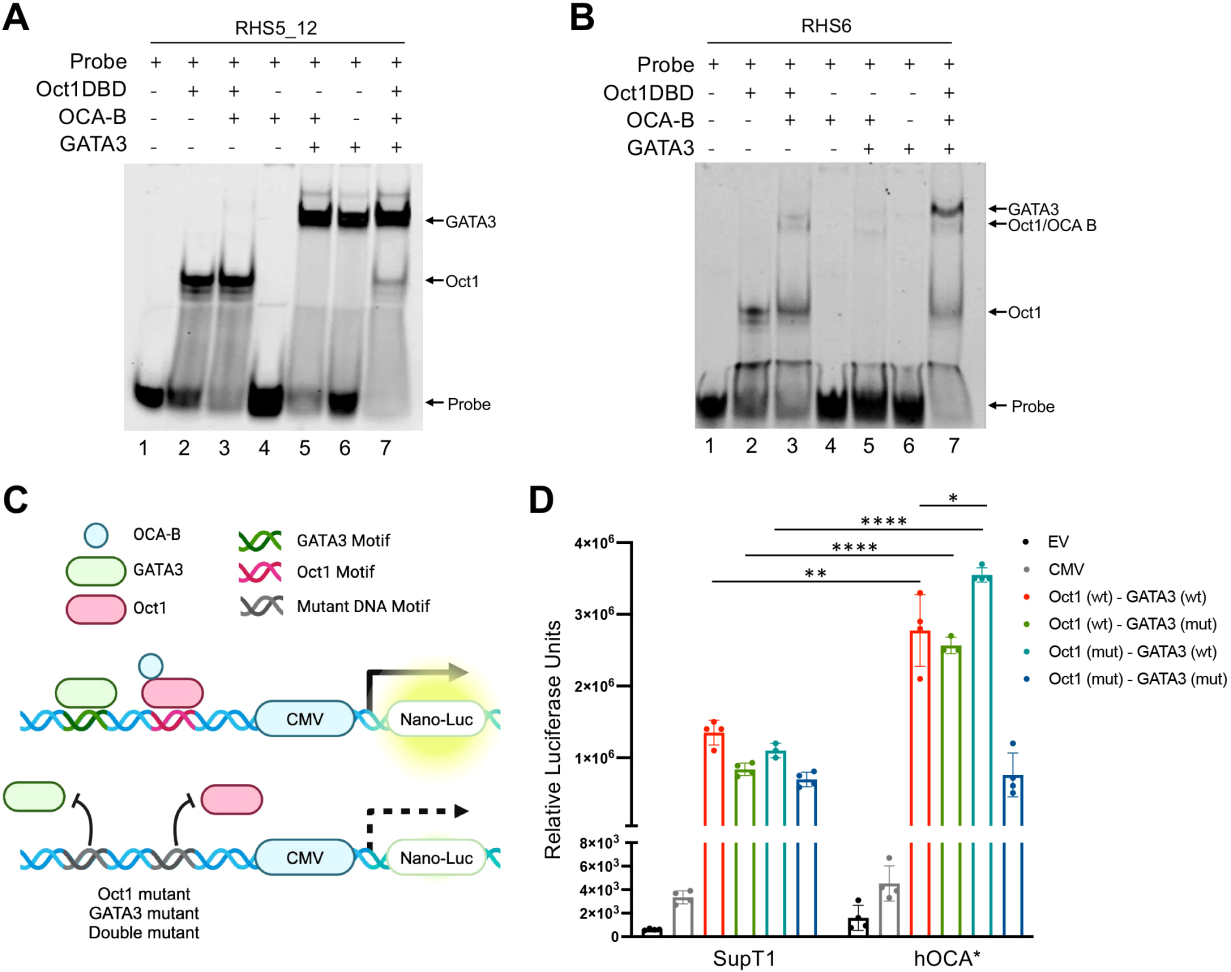
OCA-B restricts gene expression at the Th2 LCR. **(A)** EMSA showing Oct1/OCA-B/GATA3 binding to Cy5-labeled RHS5_12 DNA probe. Free probe and protein:DNA associations are indicated with arrows. **(B)** EMSA showing Oct1/OCA-B/GATA3 binding to Cy5-labeled RHS6 DNA probe. Probe and protein DNA associations are indicated with arrows. **(C)** Schematic showing the development of the RHS5 nLuc reporter constructs. **(D)** Luciferase reporter assay showing the relative secreted luciferase activity of SupT1 cells electroporated with RHS5-nLuc reporter constructs after 24 hours. All values represent mean ±SD. ns = *p*>0.05, * = *p ≤* 0.05, ** = *p ≤* 0.01, **** = *p ≤* 0.0001.

The Th2 LCR regulates the expression of *Il4*, *Il5*, and *Il13* (29). The LCR is located at the 3’ region of the *Rad50* gene and includes DNase I hypersensitive sites RHS4-7, which are necessary for efficient Th2 gene expression (30). In Th2 cells, GATA3 and Oct1 have been shown to bind the RHS5, RHS6, and RHS7 hypersensitive sites (12, 26, 31), yet how OCA-B influences transcription at the Th2 locus remains unknown. To determine if OCA-B regulates Oct1 and GATA3 binding to the Th2 LCR, we employed electrophoretic mobility shift assays (EMSA) using RHS5 and RHS6 DNA probes containing both Oct1 and GATA3 DNA binding sites. Both purified Oct1 DNA binding domain (Oct1-DBD) and GATA3 efficiently bound the RHS5 and RHS6 DNA probes, however OCA-B only bound and supershifted Oct1-DBD bound to the RHS6 probe (**Figures 3A, B**). The difference is likely due to the ATGCAAAG Oct1 binding site in RHS6 and ATTAGCAT site in RHS5, which are known to differ in their ability to recruit OCA-B *in vitro* (32). OCA-B was unable to supershift GATA3 bound to either the RHS5 or RHS6 probe, further suggesting OCA-B does not directly interact with GATA3. Similarly, no Oct1/GATA3 co-occupancy was observed in limiting probe conditions, suggesting that Oct1 and GATA3 compete for RHS5 and RHS6 probe binding.

To evaluate if OCA-B can influence transcriptional at the Th2 LCR, we cloned the wild-type RHS5 DNA fragment or versions with mutated Oct1 and GATA3 motifs upstream of a CMV promoter driving expression of secreted nano-luciferase (**Figure 3C**). Parental and hOCA* SupT1 cells engineered with an OCA-B deletion were electroporated with either control (EV and CMV-Only) or RHS5 experimental nano-luciferase reporter constructs and evaluated for secreted luciferase activity after 24 hrs. Compared to the CMV-only control construct, parental SupT1 cells showed a large increase in luciferase expression with the addition of the RSH5 enhancer (**Figure 3D**). Enhancer activity was diminished upon either Oct1 or GATA3 motif mutation. Interestingly, hOCA* SUPT1 cells showed a significant increase in luciferase expression compared to parental cells (**Figure 3D**), consistent with a model in which OCA-B restricts transcription at RHS5. Furthermore, hOCA* cells showed an elevated luciferase expression when the Oct1 motif was mutated, suggesting that OCA-B may also restricts GATA3 transcription independent of Oct1 (**Figure 3D**).

### 3.4 T cell OCA-B diminishes the Th2 immune response *in vitro* and *in vivo*

**Figure 4:**
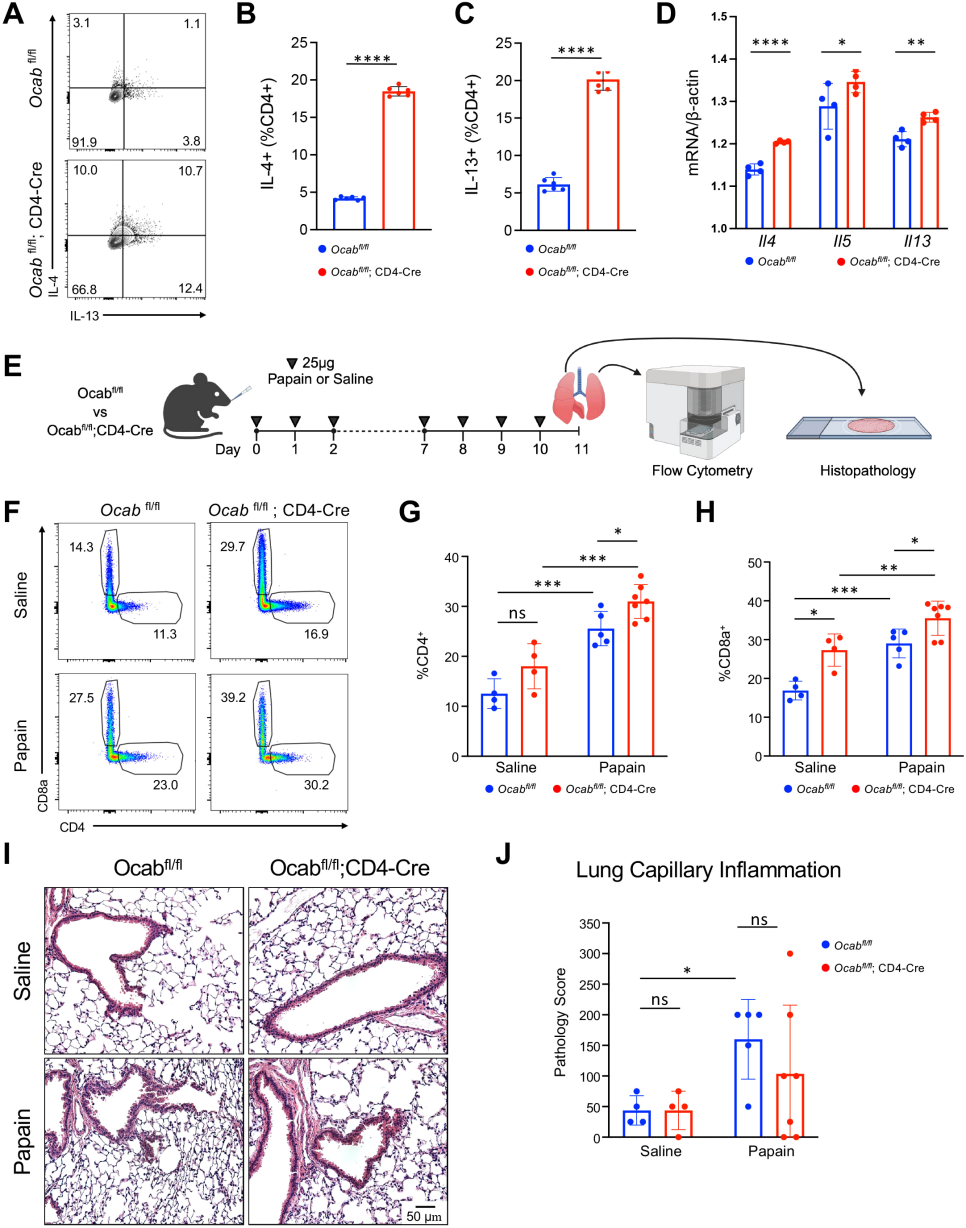
T cell OCA-B diminishes the Th2 immune response *in vitro* and *in vivo*. Splenic naïve CD4^+^ T cells were isolated from *Ocab^fl/fl^* (n=6) and *Ocab^fl/fl^*;CD4-Cre (n=6) mice and differentiated *in vitro* into Th2 cells. **(A)** Representative flow cytometry plot showing the frequency of IFNγ and IL-4 expression by CD4^+^ Th2 differentiated cells isolated from *Ocab^fl/fl^* and *Ocab^fl/fl^*;CD4-Cre mice. **(B)** Quantification of the frequency of IL-4 expression by CD4^+^ Th2 differentiated cells isolated from *Ocab^fl/fl^* and *Ocab^fl/fl^*;CD4-Cre mice. **(C)** Quantification of the frequency of IL-13 expression by CD4^+^ Th2 differentiated cells isolated from *Ocab^fl/fl^* and *Ocab^fl/fl^*;CD4-Cre mice. **(D)** qPCR showing normalized gene expression of *Il4*, *Il5*, and *Il13* in CD4^+^ Th2 differentiated cells isolated from *Ocab^fl/fl^* (n=4) and *Ocab^fl/fl^*;CD4-Cre mice (n=4). **(E)** Schematic showing dosing routine for papain allergen challenge. 10 week-old male/female littermate *Ocab^fl/fl^* and *Ocab^fl/fl^*;CD4-Cre mice were subjected to saline or papain allergen challenge, and lungs were harvested on day 11 for flow cytometric and histological analysis (*Ocab^fl/fl^* papain n=5, *Ocab^fl/fl^*;CD4-Cre papain n=7, *Ocab^fl/fl^* saline n=4, *Ocab^fl/fl^*;CD4-Cre saline n=4). **(F)** Representative flow cytometry plot showing the frequency of CD4^+^ and CD8^+^ T cells isolated from the lungs of *Ocab^fl/fl^* and *Ocab^fl/fl^*;CD4-Cre mice treated with either saline or papain. **(G)** Quantification of the frequency of CD4^+^ T cells isolated from the lungs of *Ocab^fl/fl^* and *Ocab^fl/fl^*;CD4-Cre mice treated with either saline or papain. **(H)** Quantification of the frequency of CD8^+^ T cells isolated from the lungs of *Ocab^fl/fl^* and *Ocab^fl/fl^*;CD4-Cre mice treated with either saline or papain. **(I)** Representative H&E histopathological images of *Ocab^fl/fl^* and *Ocab^fl/fl^*;CD4-Cre mice after saline or papain allergen challenge. **(J)** Pathological scoring of lung capillary tissue from *Ocab^fl/fl^* and *Ocab^fl/fl^*;CD4-Cre mice following saline or papain allergen challenge. All values represent mean ±SD. ns = *p*>0.05, * = *p ≤* 0.05, ** = *p ≤* 0.01, *** = *p ≤* 0.001, **** = *p ≤* 0.0001.

To investigate how OCA-B impacts Th2 cytokine regulation on cellular level we utilized previously reported OCA-B T cell conditional knockout mice (16). Naïve splenic T cells from control *Ocab^fl/fl^* and experimental *Ocab^fl/fl^*;CD4-Cre mice were isolated and differentiated into Th2 cells. Following differentiation, Ocab^fl/fl^;CD4-Cre Th2 cells showed elevated IL-4 and IL-13 production compared to Th2 cells from Ocab^fl/fl^ controls (**Figures 4A–C**). To determine if OCA-B regulates Th2 cytokine production on a transcriptional level we performed qPCR to evaluate expression of *Il4*, *Il5*, and *Il13*. *Il4*, *Il5*, and *Il13* mRNA transcripts were significantly elevated in Th2 cells from *Ocab^fl/fl^*;CD4-Cre mice when compared to *Ocab^fl/fl^* controls (**Figure 4D**). To determine if the loss of OCA-B in T cells alters the *in vivo* Th2 response, we induced allergic lung inflammation model using the proteolytic enzyme papain. *Ocab^fl/fl^* control and *Ocab^fl/fl^*;CD4-Cre experimental mice were intranasally inoculated with 25 μg papain or saline for 2 days, removed from treatment for 5 days, then rechallenged for an additional 3 days before lungs were harvested for analysis by flow cytometry and histology (**Figure 4E**). Flow cytometry analysis of the lung hematopoietic compartment following papain rechallenge revealed that B cells, NK cells, alveolar macrophages, cDC1, cDC2, eosinophils, monocytes, and T cell count were largely unaffected by T cell OCA-B deletion (**Supplementary Figures 2A–J**). However, the frequency of both CD4^+^ and CD8^+^ T cells were increased in *Ocab^fl/fl^*;CD4-Cre animals following rechallenge suggesting T cell OCA-B expression specifically limits T cell allergic inflammation (**Figures 4F–H**). Interestingly, the frequency of CD8^+^ T cells was increased in the *Ocab^fl/fl^*;CD4-Cre saline treated animals (**Figure 4H**). Histological analysis of lung tissue by hematoxylin and eosin staining showed an increase in lung capillary inflammation associated with the papain treatment group, indicating that the protocol successfully elicits inflammation, however no differences were observed in the OCA-B T cell knockout compared to control groups (**Figures 4I, J**). This indicates that although OCA-B loss augments T cell frequency following papain treatment, these differences are insufficient to alter lung inflammation generally.

## 4 Discussion

A detailed understanding of the transcriptional mechanisms underlying CD4^+^ T cell lineage commitment, plasticity, and effector function is necessary to reveal how CD4^+^ T cells play diverse roles in health and disease. The lymphocyte-specific transcriptional coregulator OCA-B is expressed in CD4^+^ T cells following stimulation, where it docks with Oct1 to poise immunomodulatory genes for robust expression upon repeated stimulation (13, 14). Beyond the OCA-B interaction with Oct1 and histone demethylase Jmjd1a, little is known about OCA-B protein interactions in T cells. Here, we identified 66 novel OCA-B associations in T cells by proximity labeling, including 11 isoform-specific and 22 pan-isoform protein associations. Notably, we observed multiple components of the SWI/SNF complex including SMARCA2, SMARCB1, BCL7B/C, and ARID1A. SWI/SNI components have been associated with both OCA-B and the OCA-B paralog OCA-T in non-T cells (33). One protein significantly enriched using all OCA-B isoforms was the transcription factor GATA3, which is essential for Th2 cell differentiation and function (34, 35). GATA3 controls Th2 cytokine expression by binding the promoters of *Il4*, *Il5*, and *Il13* and other regulatory elements including RHS5–7 of the Th2 LCR (29, 30). Despite enrichment in the BioID screen, no stable interaction between OCA-B and GATA3 was observed, suggesting instead a regular proximal association or possible transient interaction between the proteins.

Previous studies have shown that Oct1 can bind the Th2 LCR and synergistically activate Th2 gene expression alongside Gata3 (26), however the broader extent of Oct1/Gata3 colocalization remained unclear. To determine if OCA-B and Gata3 regularly colocalize on DNA we evaluated existing ChIP-seq data, however because OCA-B antibodies perform poorly in ChIP-seq (14), we used Oct1 as a proxy. Re-analyzing T cell ChIPseq datasets, we observed coenrichment and spatial correlation between Oct1 and Gata3, highlighting a probable mechanism behind proximity labeling of Gata3 by OCA-B. Oct1 and Gata3 coenrichment was extensive at Th2 modulatory genes regulatory regions including *Gata3*, *Irf4*, and RHS5–6 of *Rad50* of the Th2 LCR.

Purified Oct1-DBD and GATA3 protein were able to bind DNA probes from the RHS5 and RHS6 locus, but OCA-B failed to bind and supershift the GATA3 bands. OCA-B only supershifted the Oct1-DBD bound to the control octamer and RHS6 probes, supporting previous research indicating that OCA-B requires an adenine in the 5^th^ position of the octamer motif to facilitate binding *in vitro* (32). This base is missing in the RHS5 probe. The ability of OCA-B to distinguish this base position is mostly restricted to *in vitro* assays, as ChIP-seq in cells shows a broader binding pattern to noncanonical octamer sequences lacking an A at position 5 (14). Notably, Oct1 and GATA3 were unable to bind the same DNA probe, strongly suggesting that these proteins compete for DNA binding at these RHS5 and RHS6 regions. Reporter assays utilizing SupT1 cells and the RHS5 sequence showed that transcriptional activity was augmented by OCA-B disruption. Additionally, reporter activity in hOCA* cells was elevated when the Oct1 binding site was disrupted. The latter finding suggests that OCA-B restricts transcription from RHS5 through a mechanism operating independently of Oct1 binding to this motif. Oct1 and GATA3 are known to interact with the lysine-specific demethylase UTX (36, 37), which was also enriched in our OCA-B BioID. It is possible that OCA-B assists in the preferential recruitment of UTX to Oct1 at Th2 genomic regions, sequestering UTX from GATA3 and limiting GATA3 mediated transcription. Oct1 and OCA-B can also induce the expression of Bcl6 which can in turn repress GATA3 expression (38–41), suggesting another possible mechanism by which OCA-B could repress Th2 gene expression.

Previous *in vitro* and *in vivo* studies have suggested that OCA-B promotes Th1 and Th17 cellular programs while inhibiting Th2 programs (15, 42). *In vitro* Th2 differentiation experiments showed that OCA-B T cell deficiency transcriptionally elevates *Il4*, *Il5*, and *Il13* production, suggesting a T cell-intrinsic role for OCA-B inhibition of Th2 gene expression. These data were supported by an *in vivo* allergen challenge of OCA-B T cell conditional knockout mice which showed an increased frequency of T cells within the lung. Cumulatively, the results indicate that OCA-B expression in T cells can restrict Th2-associated transcription, cytokine production, and augment type-2 immune responses.

## Data Availability

Mass spectrometry data from the OCA-B BioID screen has been deposited to the UCSD Center for Computational Mass Spectrometry database (MSV000096764). ChIPseq datasets are located on NCBI’s GEO database under accession codes GSM1611108 (Oct1) and GSM742023 (Gata3).
