# Supplementary material for: Transcriptional co-regulator OCA-B/Pou2af1 restricts Th2 differentiation
Source: Front Immunol. 2025 Apr 29;16:1548636. doi: 10.3389/fimmu.2025.1548636 (PMC12069319; doi:10.3389/fimmu.2025.1548636)
Supplement: Supplementary file 1 [file DataSheet1.pdf]

Supplementary Figures

Transcriptional co-regulator OCA-B/Pou2af1 restricts Th2 differentiation

Erik P. Hughes, Asit K. Manna, Wenxiang Sun, Sandra M. Osburn-Staker, Kristi J. Warren, James E. Cox, Dean Tantin

Corresponding author: Dean Tantin, dean.tantin@path.utah.edu

Supplementary figures

Supplementary Figure 1

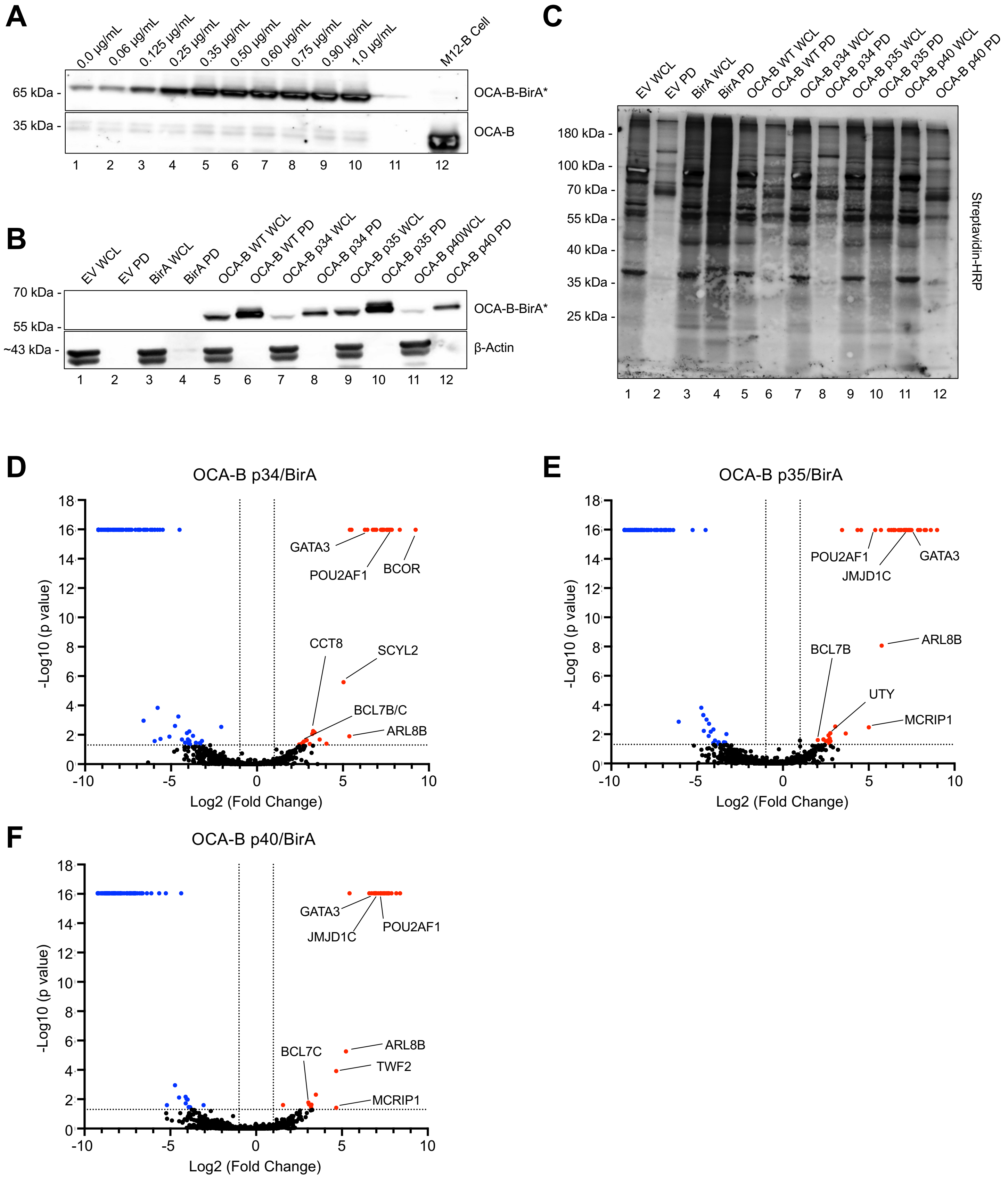

Supplementary Figure 1 OCA-B isoform BioID validation and protein enrichment.

(A) Immunoblot showing endogenous OCA-B and OCAB-BirA fusion protein expression following incubation of cells with different concentrations of doxycycline. The M12 B cell line is used as a positive control for endogenous OCA-B. (B) Immunoblot showing OCA-B-BirA isoform expression in whole cell lysates (WCL) and following streptavidin pulldown (PD). Beta-actin expression represents a protein loading control. (C) Streptavidin-HRP Immunoblot showing protein biotinylation from control (EV and BirA) and OCA-B isoform WCL and PD samples. (D-F) Volcano plots depicting proteins identified by mass spectrometry positively and negatively enriched from OCA-B p34 (D, n=3), OCA-B p35 (E, n=3), and OCA-B p40 (F, n=3) transduced over the BirA-only (n=3) transduced control SupT1 cells. Proteins significantly elevated in wild-type OCA-B replicates are shown in red and proteins significantly elevated in BirA-only replicates are shown in blue.

Supplementary Figure 2

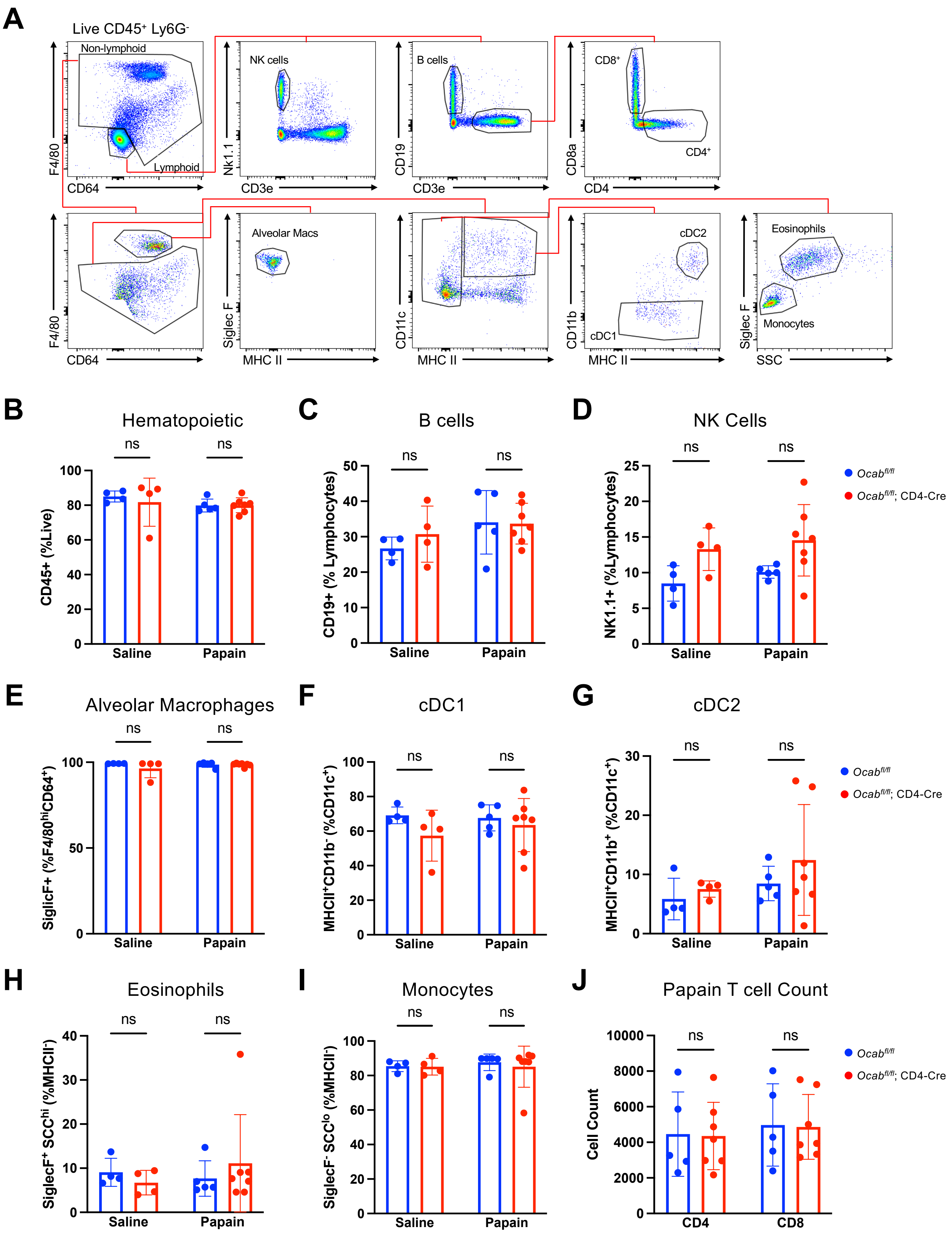

Supplementary Figure 2 Lung flow cytometry analysis following papain challenge.

Flow cytometry antibodies were purchased from Becton-Dickinson (CD4-BUV395, CD19-BUV661, CD8a-BUV737, IA/IE-BUV805) and BioLegend (Ly6G-PB, CD64-BV711, SiglecF-FITC, CD45-Percp, CD11c-Percp-cy5.5, NK1.1-PE-Cy5, F4/80-APC, CD3e-PE/Dazzle594, and CD11b-APC/Cy7). (A) Representative flow cytometry gating strategy to evaluate immune cell subsets isolated from lung tissue. (B-I) Quantification of the frequency of hematopoietic ($CD45^+$), B cells ($CD19^+$), NK cells ($NK1.1^+$), alveolar macrophages ($SiglicF^+$, $F4/80^{hi}$, $CD64^+$), cDC1 cells ($MHCII^+$ $CD11b^-$, $CD11c^+$), cDC2 cells ($MHCII^+$ $CD11b^+$, $CD11c^+$), eosinophils ($SiglicF^+$, SSC^{hi}, $MHCII^-$), and monocytes ($SiglicF^-$, SSC^{lo}, $MHCII^-$) isolated from the lungs of $Ocab^{fl/fl}$ and $Ocab^{fl/fl}$;CD4-Cre mice treated with either saline or papain. (J) Quantification of cell counts from $CD4^+$ and $CD8^+$ T cells isolated from the lungs of $Ocab^{fl/fl}$ and $Ocab^{fl/fl}$;CD4-Cre mice treated with papain. All values represent mean ±SD. ns = $p>0.05$, * = $p\leq0.05$, ** = $p\leq0.01$, *** = $p\leq0.001$, **** = $p\leq0.0001$.
